# Silicon Oxycarbide Porous Particles and Film Coating as Strategies for Tenofovir Controlled Release in Vaginal Tablets for HIV Prevention

**DOI:** 10.3390/pharmaceutics14081567

**Published:** 2022-07-28

**Authors:** Araceli Martín-Illana, Raúl Cazorla-Luna, Fernando Notario-Pérez, Roberto Ruiz-Caro, Juan Rubio, Aitana Tamayo, María Dolores Veiga

**Affiliations:** 1 Department of Pharmaceutics and Food Technology, Faculty of Pharmacy, Universidad Complutense de Madrid, Plaza Ramón y Cajal s.n, 28040 Madrid, Spain; aracelimartin@ucm.es (A.M.-I.); racazorl@ucm.es (R.C.-L.); rruizcar@ucm.es (R.R.-C.); mdveiga@ucm.es (M.D.V.); 2 Institute of Ceramics and Glass, CSIC, Kelsen 5, 28049 Madrid, Spain; jrubio@icv.csic.es (J.R.); aitanath@icv.csic.es (A.T.)

**Keywords:** AIDS, antiviral therapy, chitosan, hydroxypropyl methylcellulose, mucoadhesion, pre-exposure prophylaxis, sustained drug release, women’s health

## Abstract

Sustained release of antiretroviral drugs is currently the most encouraging strategy for the prevention of the sexual transmission of HIV. Vaginal tablets based on hydrophilic gelling polymers are an interesting dosage form for this purpose, since they can be developed to modify the release of the drug depending on the tablet swelling. Tenofovir is a drug with proven activity in the prevention of HIV-1 infection, and it is possible to have it loaded in the surface of γ-aminopropyl trimethoxy silane-functionalized oxycarbide particles. These particles can be incorporated into the tablets, thus providing a sustained release of the drug. Moreover, the presence of the particles modifies the microstructure of the gel formed, as observed in scanning electron microscopy and Hg porosimetry studies, resulting into a gel with a narrow pore size distribution between 10 and 100 µm. This implies a lower volume of fluid incorporated into the gel during swelling studies, and therefore improved mucoadhesion times in ex vivo test. The coating of the formulations with Eudragit^®^ RS modifies the swelling behavior of the tablets, which not only is decreased in magnitude but also extended in time, and as consequence the drug release is also prolonged for up to 7 days.

## 1. Introduction

In the last International Women’s Day, the Executive Director of UNAIDS highlighted the need to empower women to end the AIDS pandemic. The infection by HIV represents the third leading cause of death in women between the ages of 15 and 49, and only 55% of women report that they have control over decisions about their sexual and reproductive health and rights [1].

Among the different available tools for HIV prevention that could be under women’s control, vaginal microbicides can be highlighted. They are agents for topical application intended to pre-exposure prophylaxis by blocking the initial stages of the infection in the vagina. Gels, capsules, tablets, films, and intravaginal rings are some of the developed systems for this purpose [2]. Among them, vaginal tablets offer several advantages, such as easy and economical manufacture at the industrial scale, versatility in the formulation in terms of drug release control, easy handling and stability in different environmental conditions [3]. Several vaginal tablets intended for the prevention of HIV have already reached the clinical phase. For example, vaginal tablets containing the reverse transcriptase inhibitors Tenofovir (TFV) and/or Emtricitabine [4] and an entry inhibitor named DS003 [5] have been tested in clinical trials.

As stated by Cobb et al. [6], most microbicides are designed for on-demand use and offer short-acting protection against the virus. For this reason, the development of controlled-release antiretroviral formulations could solve the problem of acceptability and adherence of these prevention systems. Against immediate drug release, drug controlled release offers advantages such as lower side effects and improved compliance by patients due to reduced administration frequency [7]. This type of drug release can be achieved by including one or more excipients capable of controlling the release process in the formulation. Among them, it is worth mentioning swellable polymers, whose structure is modified in the presence of water or biological fluids, thus altering the release of the drug to the medium. One of the most frequent polymers in the development of swellable matrices is hydroxypropyl methylcellulose (HPMC) [8], a semi-synthetic non-ionic cellulose derivative with excellent mucoadhesive characteristics [9]. Another polymer is chitosan, a chitin-derivative polysaccharide which exhibits mucoadhesive properties due to its cationic nature. It also has swelling ability and a huge potential for controlled drug release [10]. The combination of HPMC and chitosan as mucoadhesive polymers in the formulation of vaginal tablets for the controlled release of TFV has already allowed to enhance the properties offered by the systems composed by each polymer separately [11].

Another type of excipients that has been widely studied for controlled drug release is porous materials. Properties such as their well-organized structure, high surface area, and pore sizes which can be tuned make them excellent candidates as drug carriers [12]. According to the pore size, porous materials can be classified as microporous (<2 nm), mesoporous (2–50 nm), or macroporous (>50 nm). In addition, they can be organic, inorganic, or hybrid in nature [13]. Mesoporous silica- and carbon- based materials could be cited among the inorganic mesoporous materials that have been most used as drug carriers [12,14]. In this line, we previously synthesized mesoporous silicon oxycarbide particles as an excipient for the controlled release of Acyclovir [15] and micro-mesoporous hybrid particles for the sustained release of TFV [16]. The developed particles were evaluated in terms of cellular toxicity using two human cell lines: HEC-1-A (a uterus/endometrium epithelial cell line) and MT-2 (a lymphoblastic cell line). The MTT method was used to evaluate cell toxicity, which was recorded after 48 h of incubation of the cells in the presence of the inorganic particles. The results ensure the safety of the inorganic material, that did not present cytotoxic effect in the concentrations tested (up to 1 mg/mL) [15].

Finally, the coating of solid dosage forms represents a broadly used strategy to obtain a controlled drug release. Film coating is the most common and versatile technique and entails the application of a polymeric formulation on the surface of solid dosage forms such as tablets. This resource allows not only to improve characteristics such as the appearance and organoleptic properties of the dosage forms, but also to modify the release of the drug [17]. Polymethacrylates, including those commercialized under the Eudragit^®^ brand, are synthetic copolymers which exhibit great film-forming properties. They are frequently used as pharmaceutical coating excipients to obtain drug controlled release profiles, among others [18]. Namely, Eudragit^®^ RS (ammonium methacrylate copolymer type B), which presents quaternary ammonium groups in its structure, is characterized by being insoluble in water but permeable, which allows the controlled release of drugs when used in film coating [19]. In addition to providing sustained drug release, it also has adhesive properties; a study showed that nanocapsules based on Eudragit^®^ RS were more mucoadhesive than those manufactured with Eudragit^®^ S and Poly(ε-caprolactone) [20].

With this background, the objective of the present research work was to develop TFV-controlled release mucoadhesive vaginal tablets by combining the three aforementioned strategies (mucoadhesive swellable polymers, inorganic particles and film coating) for the prevention of the sexual transmission of HIV in women.

## 2. Materials and Methods

### 2.1. Materials

Triethoxysilane (TREOS, 99%), isopropanol (iPrOH, 99%), HCl (35%), and NH_4_OH (28%) were acquired from Merck (Darmstadt, Germany). Hydroxyl terminated polydimethyl siloxane (PDMS, M = 1700 g·mol^−1^) and γ-aminopropyl trimethoxy silane (APS, 98%) were supplied by ABCR (Karlsruhe, Germany) and Gelest (Morrisville, PA, USA), respectively.

Chitosan (MW = 10^5^ g/mol, deacetylation degree = 97% and viscosity = 92 mPa·s [21]) was supplied by Nessler (Madrid, Spain) and hydroxypropyl methylcellulose Methocel^®^ K 100 M (HPMC, MW = 72 × 10^4^ g/mol) was a kind gift from Colorcon Ltd. (Kent, UK). Kollidon^®^ 30 (polyvinyl pyrrolidone K30, PVP) and magnesium stearate PRS-CODEX (MgSt) were supplied by BASF (Ludwingshafen, Germany) and Panreac (Barcelona, Spain), respectively. Tenofovir (TFV) was acquired from Carbosynth Limited (Compton, UK). Eudragit^®^ RS (ERS, MW = 407.932 g/mol) was kindly supplied by Evonik (Essen, Germany). Triethyl citrate (TEC) and acetone were acquired from Sigma-Aldrich^®^ (St. Louis, MO, USA) and Panreac (Barcelona, Spain), respectively.

All other reagents in this study were of analytical grade and used without further purification. Demineralized water was used in all cases.

### 2.2. Obtaining, Functionalization and TFV-Loading of Silicon Oxycarbide (SiOC) Particles

**Figure 1:**
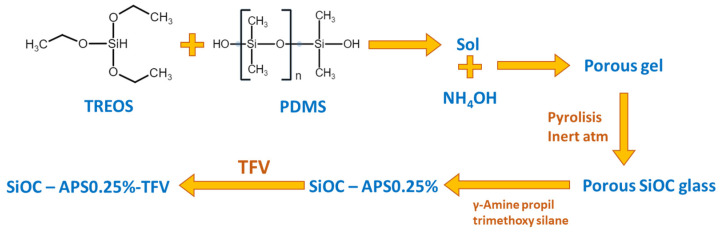
Scheme of the synthesis of γ-aminopropyl trimethoxy silane-functionalized oxycarbide particles (APS-SiOC).

The synthesis of the SiOC material based on the sol-gel method was previously described (Figure 1) [15]. Briefly, two solutions were separately prepared and stirred for 2 h until homogenization; the first containing TREOS and PDMS in 70/30 weight ratio and ½ iPrOH and the second with H_2_O, HCl and ½ iPrOH. The H_2_O/iPrOH/HCl/TREOS molar ratio was 3/4.5/0.05/1. The second solution was added dropwise over the first one and thermostatized at 80 °C for 2 h. Within the first minutes after the beginning of the reaction, 1 M NH_4_OH solution was added (in a 1:2 volume proportion with respect to the wet gel) for the resulting sol to be gelled. The obtained gel was dried at 50 °C for a week and then at 120 °C until constant weight. After that, it was pyrolyzed at 1100 °C for 2 h in N_2_ atmosphere giving rise finally to a porous SiOC material.

Once obtained, the SiOC material was functionalized with APS in aqueous medium. The coupling agent (APS) was previously hydrolyzed for 30 min in water at 25 °C at a 0.25% APS/H_2_O w/w ratio. After that, 0.5 g of SiOC particles were added and the stirring was maintained for an additional 30 min. The resulting surface modified material was filtered, dried at 50 °C overnight and then at 110 °C for 6 h.

The APS-functionalized oxycarbide particles were then loaded with TFV. First, 300 mg of the drug was dissolved in 220 mL of water under stirring for 2 h. After that, 0.45 g of functionalized SiOC particles were added and the mixture was stirred for 30 min and finally placed in a glycerin bath at 50 °C until the complete evaporation of the solvent (2 days).

### 2.3. Tablets Manufacture

Three batches of tablets containing the previously obtained TFV-loaded SiOC particles were prepared. Their composition is shown in Table 1. The difference between these batches is the chitosan/HPMC ratio, which is 1 for T1, 0.53 for T2, and 1.9 for T3. In all cases, the required amounts of TFV-loaded particles for a dose of 30 mg of drug were used. The manufacturing of the tablets was based on previously described methodologies [11,22]. Briefly, chitosan, HPMC, and TFV-loaded SiOC were physically mixed. After that, a wet granulation using a 0.5 mm mesh metal sieve and an ethanolic solution of PVP as a binder was performed. The obtained granulate was dried at room temperature for 24 h and then the corresponding amount of MgSt was added. The flowability of the granules was determined according to the methodology described in the European Pharmacopoeia (2.9.16) and compared to the flowability of the powders before granulation. Finally, the granules were compressed using a press like that used in the preparation of IR spectroscopy pills. The force applied was 5 t for 4 min for each tablet. Thickness, diameter, and hardness of all the batches were measured in triplicate using a hardness tester PTB 311 (Pharma Test, Hainburg, Germany).

### 2.4. Coating of the SiOC-Based Tablets

The previously manufactured tablets were subsequently coated to increase the control over drug release and improve some other properties, such as comfortability in terms of swelling. The composition of the coated tablets is collected in Table 2. A solution of Eudragit^®^ RS (10% *w*/*v*) in acetone was used as coating solution. About 0.5% *w*/*v* TEC was included as plasticizer in the coating solution. Each tablet was immersed twice in this solution and left to dry for 24 h at room temperature. The process was repeated until the total coating supposed an increase of around 10% (7–11%) in the initial weight of the tablet.

### 2.5. Characterization Techniques

#### 2.5.1. Swelling Test

This test was performed to evaluate the influence of the vaginal fluid in the structure of the tablets due to the presence of the swellable polymers (chitosan and HPMC) and how this process could condition the drug release. Based on a method described by Mamani et al. [23], three tablets of each batch (both coated and uncoated) were fixed to stainless steel discs of 3 cm diameter using ethyl cyanoacrylate (Loctite^®^) as adhesive material. Each disc was then immersed in a beaker containing 100 mL of simulated vaginal fluid (SVF, pH = 4.2 [24]), then placed into an oscillating water bath (P SELECTA^®^ UNITRONIC OR, JP SELECTA S.A., Barcelona, Spain) at 15 opm and 37 °C, thus simulating the in vivo conditions. At preset times, the discs with the formulations were extracted from the beakers and weighted on a precision balance (METTLER^®^ AT 200, Mettler-Toledo S.A.E., Barcelona, Spain) after removing the excess of SVF using a paper towel. The swelling ratio was calculated according to the following Equation (1):

(1) $$Swelling\;ratio\;\left(\%\right)=\frac{T_{s}-T_{d}}{T_{d}}\times 100$$

where *T_s_* refers to the weight of the swollen tablet at each weighing time and *T_d_* to the weight of the tablet before the immersion in SVF (dry).

As the weight of the tablets is not the same in all the batches but the amount of swellable polymer is, for a correct comparison between coated and uncoated tablets, the adjusted swelling ratio was calculated using the Equation (2):

(2) $$Adjusted\;swelling\;ratio=\frac{SR\cdot T_{d}}{SP}$$

Being *SR* the previously calculated swelling ratio, *T_d_* the weight of the tablet and *SP* the amount of swellable polymer in the tablet.

At the same predetermined weighing times, the diameter of the swollen tablets was measured to observe how the volume of the formulation is modified as a function of the swelling.

The consistency of the swollen tablets was measured by means of a TA.TXT*plus* Texture Analyzer at 2, 6, 48, and 120 h. A protocol was designed with the equipment on compression mode, loaded with a 5 kg load cell, using a cylindrical probe (5 mm) moving at 0.5 mm/s. Penetration force and work were recorded. The height of the gel and the dried tablet were extrapolated from the results.

#### 2.5.2. Scanning Electron Microscopy (SEM) and Hg Porosimetry

To determine how the inclusion of the SiOC particles and the arrangement of the polymer chains define the structure of the tablets in the presence of vaginal fluid, the uncoated swollen tablets were studied by SEM and mercury porosimetry. The dry tablets were previously attached to the stainless-steel discs and then immersed in beakers containing 100 mL of SVF, which were placed in the oscillating water bath at 37 °C and 15 opm, as described in the swelling test. Once the formulations reached their maximum swelling ratio, they were extracted from the medium and freeze-dried in a Lio-Labor^®^ freeze dryer (Telstar, Barcelona, Spain) with a freezing temperature of −45 °C, a sublimation temperature from −45 to 25 °C and a sublimation pressure of 4.54 × 10^−4^ atm attained inside the chamber. The obtained freeze-dried samples are suitable only for comparative structure analysis.

The microstructure of the resulting freeze-dried tablets was observed using a field emission scanning electron microscope (Hitachi S-4700, Tokyo, Japan) at 20.0 kV and mercury porosimetry was carried out with an Autopore II 9215 (Micromeritics Corp., Norcross, GA, USA) to determine pore size distributions (PSD). The corresponding pore volumes (Vp), pore areas (Sp), mean pore sizes (Dp), bulk and apparent densities (ρB, ρA), and porosities (P) of the swelling witnesses were calculated from these PSD, assuming cylindrical pore shapes.

#### 2.5.3. Mucoadhesion Test

The adhesion time of the tablets to the mucosa was evaluated by an ex vivo study based on a previously described methodology [21]. A sample of bovine vaginal mucosa—kindly provided by a local slaughterhouse—was fixed to a stainless-steel plate with a width of 5 cm and a height of 8.5 cm using ethyl cyanoacrylate (Loctite^®^) as adhesive material. Subsequently, a tablet was placed on that mucosa, ensuring its adhesion by placing a weight of 500 g for 30 s to standardize the process. Each plate was fully immersed—at an angle of 60°—in a beaker containing SVF, and then into the oscillating water bath at 37 °C and 15 opm. The times required for the tablets to dissolve, erode, and/or detach from the mucosa were recorded. These assays were performed in duplicate for each formulated batch.

#### 2.5.4. Drug Release Test

One of the main objectives of this work was to obtain vaginal dosage forms offering a controlled release of TFV. Drug release test was carried out in the oscillating water bath at 37 °C and 15 opm, wherein the formulations were placed into borosilicate glass flasks containing 80 mL of SVF (thus ensuring sink conditions). Aliquots of 5 mL were extracted from each flask at preset times, replacing the volume removed with clean SVF. After filtration and dilution of the taken samples, the amount of TFV in the medium was quantified by UV-Visible spectroscopy at a wavelength of 261 nm using a Shimadzu^®^ UV-1700 spectrophotometer (Kyoto, Japan). These assays were performed in triplicate for each batch of tablets, both uncoated and coated. Similarity factor f_2_ was used to compare the drug release profiles obtained [25].

In order to understand the mechanisms responsible for the release of the drug from the SiOC particles and the tablets, the results obtained in this test were processed to determine whether they fitted the Higuchi, Hopfenberg and Korsmeyer–Peppas kinetics [26].

##### Higuchi Kinetics

It can be summarized by the following Equation (3), which is known as the “simplified Higuchi model”:

(3) $$Q_{t}=K_{H}t^{1/2}$$

where *Q_t_* is the amount of drug released at time *t* and *K_H_* is the Higuchi dissolution constant. Based on this model, the drug is released by a diffusion process according to Fick’s first law and proportionally to the square root of time.

##### Hopfenberg Kinetics

This release kinetics responds to the following Equation (4):

(4) $$\frac{M_{t}}{M_{\infty }}=1-{\left[1-\frac{k_{0}t}{C_{0}a_{0}}\right]}^{n}$$

where *M_t_/M_∞_* is the fraction of drug dissolved in the medium (*M_t_* is the amount of drug dissolved at time *t* and *M_∞_* is the dose), *k*_0_ is the erosion constant, *C*_0_ is the initial concentration of the drug in the dosage form, *a*_0_ is the radius of the sphere or cylinder or the average thickness of the slab (depending on the formulation), and *n* is the exponent which varies according to the geometry (with a value of 1 for slabs, 2 for cylinders and 3 for spheres). Considering that $k_{1}=\frac{k_{0}}{C_{0}a_{0}}$, the above equation converts into Equation (5):

(5) $$\frac{M_{t}}{M_{\infty }}=1-{\left[1-k_{1}t\right]}^{n}$$

According to this model, the drug release is caused by an erosive process of the dosage form, which can take different geometric forms as mentioned.

##### Korsmeyer–Peppas Kinetics

In general, this kinetic responds to the following Equation (6):

(6) $$\frac{M_{t}}{M_{\infty }}=at^{n}$$

where *M_t_/M_∞_* is the fraction of drug released in relation to the dose, *a* is a constant that depends on the structural and geometric characteristics of the dosage form, *t* is the time, and *n* is the exponent indicating the mechanism responsible for the drug release. In this case, diffusion predominates when the value of *n* is less than or equal to 0.45; values between 0.45 and 0.89 indicate an “anomalous transport” based on diffusion and the structural modification of the dosage form; *n* values equal to 0.89 (“case II transport”) and over 0.89 (“super-case II transport”) indicate drug releases that are due only to structural changes in the formulation.

## 3. Results and Discussion

### 3.1. SiOC Particles and Tablets Manufacture

The SiOC particles were previously characterized [15]. Summarizing, they show an irregular appearance with a rough surface, and a particle size between 10 and 15 µm. Regarding the pores in the structure of the particles, they present a bimodal PSD in the range of mesopores; there are smaller pores which have a diameter around 6 nm and bigger ones, in a size around 70 nm.

**Figure 2:**
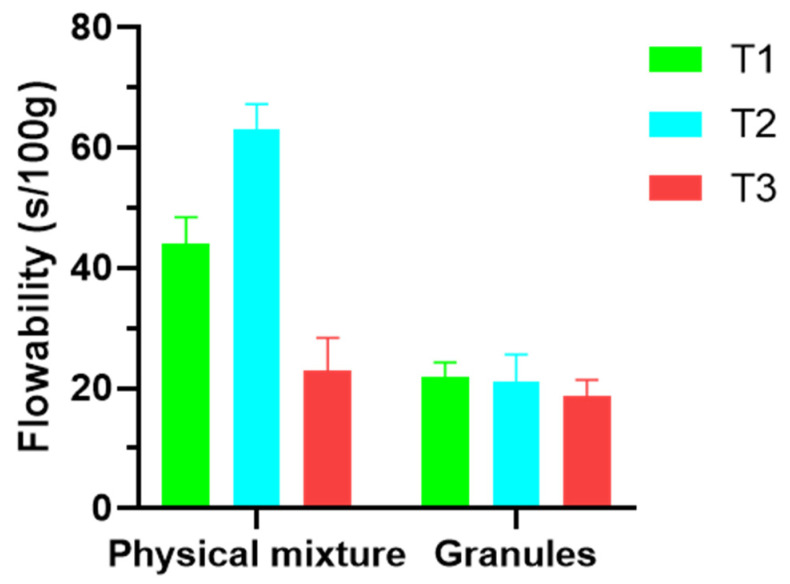
Flowability of the blends for compression before and after granulation.

The flowability of the granules is represented in Figure 2 and compared to the results obtained with the physical mixture of the powders before granulation. As can be observed, the more HPMC the mixture contains, the poorer the flowability, with batch T2 showing an average flowability of 63 s/100 g. However, it is confirmed that the granulation and the incorporation of magnesium stearate as lubricant improve the flowability of the mixtures up to 21 s/100 g.

The obtained tablets were cylindrical in shape and had a diameter of 13 mm and a height of 2.4 to 2.6 mm. Geometrical parameters, hardness, and porosity of the tablets are summarized in Table 3. As can be observed, there are no significant differences in the diameter of the batches. The hardness increases when one of the two polymers prevails over the other (mainly when chitosan predominates, with values of hardness around 340 N). Anyway, it is remarkable that high values of hardness are observed in all the batches, which is interesting from a drug release point of view, since these tablets are not intended to suffer a disintegration but to swell and act as matrix tablets. Moreover, there is a direct relation between the amount of HPMC and the porosity of the tablets, although all of them have low porosity values.

### 3.2. Characterization of SiOC-Based Tablets

#### 3.2.1. Swelling Test

**Figure 3:**
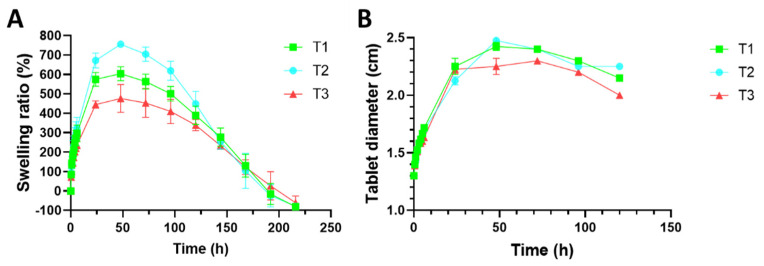
Swelling profiles (**A**) and diameter (**B**) in SVF resulting from the evaluation of the tablets developed including TFV-loaded SiOC particles.

Swelling profiles obtained from the different tablets manufactured with TVF-loaded SiOC particles are displayed in Figure 3A. In all cases, an initial stage of fluid absorption can be observed, where the values of swelling ratio increase until the maximum value is reached after 48 h. After that, the swelling ratio decreases until the complete erosion/dissolution of the system, which happens after 216 h of test. This behavior is explained by the changes suffered by the polymers in the presence of the medium. Water-soluble polymers such as chitosan and HPMC uptake water from the medium and form a gel that finally erodes or dissolves in the medium [27].

Attending to the swelling profiles obtained, some differences can be established regarding the chitosan/HPMC ratio in the tablets. The formulation that suffers the most outstanding swelling is T2, which includes the higher amount of HPMC. This was predictable, since tablets based on HPMC have already proven to suffer a prominent water uptake when exposed to aqueous fluids [21,28]. On the other hand, tablets T3 shows the lowest values of swelling. These tablets include higher proportion of chitosan over HPMC, so the moderate swelling that is exhibited by tablets based on the chitin derivative was expected to prevail [29]. Finally, tablets T1—those combining HPMC and chitosan at equal proportion—show an intermediate swelling behavior between tablets T2 and T3. On that basis, it can be affirmed that the higher the proportion of HPMC in the tablets, the higher their swelling. This was previously observed for tablets based on chitosan and HPMC in these proportions as matrix-forming polymers [11] and can be attributed to the different nature and chain arrangement in the medium of the mentioned polymers. When comparing the tablets manufactured at this work including TFV-loaded SiOC particles with the tablets previously evaluated including the same combination of HPMC/chitosan but containing anhydrous calcium hydrogen phosphate (ACDP) as structural agent [11], it can be concluded that both the maximum swelling ratio and the time at which this maximum is reached are reduced by the inclusion of the SiOC particles in the tablets. This reveals the potential of these particles to act as structural agent in the formulation even after the gelling of the hydrophilic polymers.

Regarding the tablet diameter during the swelling process (Figure 3B), not important differences can be established among the three batches despite the differences observed in the swelling ratio. As it could be expected, the diameters of the tablets increase with the uptake of water until the maximum swelling ratio is reached at 48 h. After that, the tablets suffer a slight decrease in the diameter due to the erosion/dissolution of the dosage form in the presence of SVF.

**Figure 4:**
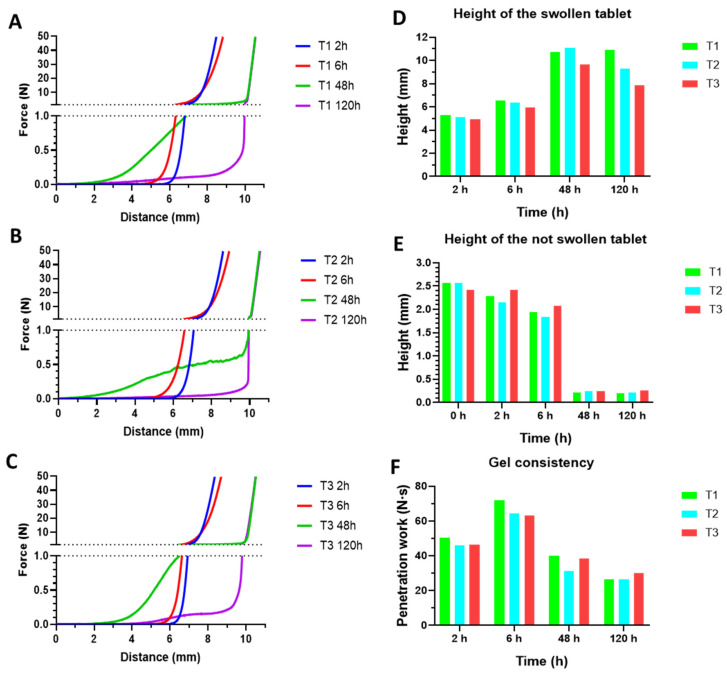
Profiles of the penetration force of swollen tablets T1 (**A**), T2 (**B**), and T3 (**C**) at 2, 6, 48, and 120 h, in SVF. Height of the formed gel (**D**), height of the dry tablet (**E**), and penetration work (**F**) at the same times.

The analysis of the consistency of the swollen tablets is represented in Figure 4A–C. Swollen tablets at 2 and 6 h show similar consistency profiles; a highly viscous gel has been formed, although it occupies low volume. At 48 h, the formed gel has lower viscosity but a higher volume, and at 120 h the volume of gel is similar, but the consistency is markedly lower. This clearly explains the layer-by-layer swelling of these tablets, where the outer layers become a gel while the core of the tablets remains not swollen.

From the data obtained in the analysis with the Texture Analyzer, it can be known the height that the formed gel has reached at the time it is analyzed (Figure 4D), as well as the size of the not swollen tablet (Figure 4E). The gel occupies a higher volume while the swelling advances, and the dry tablet is reduced accordingly until the maximum swelling is reached (48 h). From that point until the 120 h of swelling test, these parameters are barely modified. Finally, the penetration work of the probe is a measure of the gel consistency (Figure 4F). Although the maximum swelling ratio needs 48 h to be reached, the maximum consistency is observed at 6 h. From that point, the volume of the gel is increased due to the incorporation of vaginal fluid, and therefore the gel consistency is reduced.

#### 3.2.2. SEM

**Figure 5:**
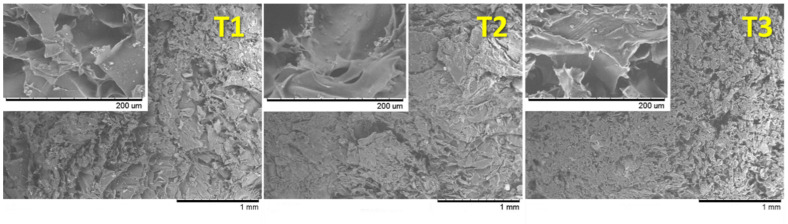
Micrographs of tablets including SiOC particles, obtained by scanning electron microscopy from freeze-dried samples at their maximum swelling.

The differences observed in the swelling behavior of the tablets were subsequently deeply studied through the analysis of the microstructure of the systems after the maximum swelling ratio is reached. SEM micrographs of freeze-dried swollen tablets are displayed in Figure 5. The gel formed from tablets T3 shows a predominantly spongy appearance after freeze-drying, although some lamellar structures can be observed at higher magnification. This is related to the presence of chitosan, which becomes a sponge-like structure after gelling [21,30,31]. On the other hand, the lamellar structure characteristic of HPMC tablets predominates in tablets T2 [21,32], that undergo a layer-by-layer swelling when exposed to an aqueous fluid [33]. Finally, tablets T1 show an intermediate appearance. The structure observed in these micrographs can be easily related to the swelling profiles obtained from each tablet: the lamellar structure attributed to HPMC allows the system to capture higher amounts of water than the sponge-like structure of chitosan—where the micropores observed after freeze-drying reveal a reduced volume that was previously filled by water. Thus, the more HPMC the tablet includes, the more it swells.

#### 3.2.3. Porosimetry

**Figure 6:**
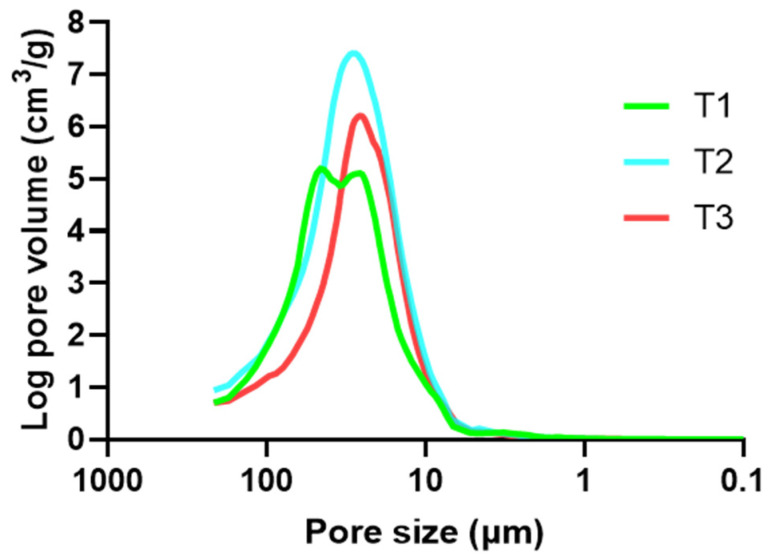
Pore size distribution (PSD) of tablets manufactured including SiOC particles, freeze-dried at the time of maximum swelling.

The characterization of the freeze-dried formulations by Hg porosimetry studies allows to know the volume of the system that is occupied by water while drug release happens, as well as the size of the pores originated in the tablet after swelling. This is a key parameter, since it can be correlated with the drug diffusion process. The pore size distributions (PSD) are represented in Figure 6.

In Figure 6, it can be observed that both formulations T1 and T2 have pores with higher diameter than formulation T3; nevertheless, while the total pore volume is similar for tablets T1 and T3, tablets T2 have a much higher total pore volume. It was previously discovered that formulations based on HPMC give rise to a gel where medium pore size is about 100 µm, while systems based on chitosan result in gels with a width PSD—between 50 and 0.5 µm—after swelling [21]. However, in the PSD displayed in Figure 6, any of the systems show pores with a lower size than 10 µm, while the pores with a size closer to 100 µm represent a minimum amount of the total pore volume. It can therefore be concluded that the mixture of HPMC and chitosan becomes a hybrid gel, where the size of the formed pores is between 100 and 10 µm. Moreover, Figure 6 shows that, while tablets T2 and T3 have a monomodal PSD, a bimodal PSD is observed in tablets T1. Although all batches have a similar medium pore size, the differences among them can reveal that, when one polymer predominates over the other, a mixed structure is formed, where the polymer included in the lower proportion is integrated into the structure of the other one. Nonetheless, when HPMC and chitosan are included at equal proportions they “compete” to form their own characteristic structure, which results in a bimodal PSD.

Another main factor that must be considered when analyzing the PSD is the presence of the TFV-loaded SiOC particles. It was already mentioned that these particles could act as a structuring agent in the swollen system, and therefore the PSD might be modified. This is confirmed when comparing the obtained PSD with those from the systems including ACDP as a structural agent instead of SiOC particles [11]. The medium pore size is notably reduced in all the formulations including the SiOC particles, which could be associated to a higher interrelationship between polymers and particles, leading to a lower available area for water entrance. There are no references in the literature about this behavior in systems including HPMC, but the increment of binding forces from cross-linking agents when chitosan was combined with hydroxyapatite has been previously described [34]. This also explains the lower total volume of vaginal fluid embedded by these systems, as observed in the swelling test; the lower the pore size, the lower the volume of fluid that can be retained.

Table 4 is a summary of the Hg porosimetry results. It allows to confirm the observations made based on Figure 6. For example, the mean pore size (Dp) is higher for tablets T1 than T2 and T3. Although the greater presence of HPMC in T2 would make expectable a higher Dp in these tablets, the result obtained can be associated to that bimodal PSD observed in tablets T1, that causes Dp to be higher. However, pore volumes (Vp) are higher for tablets T2, being T3 the ones with the lowest available volume. This result is directly related to the maximum values of swelling ratio observed in the swelling test. The values of porosity also confirm our previous observation; tablets T2 are significantly more porous than the other ones. Moreover, being the more porous one makes T2 have the lowest bulk density (ρB).

#### 3.2.4. Mucoadhesion Test

The adhesion to vaginal mucosa is required to achieve the retention of the formulation in the vagina until the drug is completely released [35]. Thus, bioadhesive formulations have proven to be a valuable tool to reduce the leakage caused by vaginal clearance and gravity effects [36]. Since SiOC particles are not expected to have adhesive properties, the evaluation of the mucoadhesiveness of the systems was crucial to verify that the natural adhesion of HPMC and chitosan is not affected by the presence of the particles. Results from mucoadhesion test are collected in Table 5. All systems showed mucoadhesion times between 144 and 168 h, which is appreciable for a controlled release vaginal system. It also highlights that those systems including a higher amount of chitosan (T3) are able to remain attached until their complete erosion in the vaginal fluid. The adhesiveness to vaginal mucosa of hybrid HPMC/chitosan tablets was previously established around 96 h [11]. Therefore, it is clear that the inclusion of TFV-loaded SiOC particles lengthens the time that the formulations can remain attached to this biological tissue. The explanation for this behavior cannot be attributed to the particles being adhesive, but to the behavior already observed in swelling studies: the presence of SiOC particles reduces the volume of vaginal fluid that is captured by the systems, which results in a double feature that is advantageous for mucoadhesion. On the one hand, the lower inclusion of water increases the time required for the polymer dissolution, and therefore hampers the erosion of the systems [37]. On the other hand, the lower volume of fluid entrapped makes the weight of the system lower, and this also benefits for longer adhesion times [38]. The denser gel implies more available polymer chains to interact with the surface of the mucosa, and therefore a higher number of interactions between the mucosa and functional groups in HPMC and chitosan—that interact with mucin by hydrogen bonds and electrostatic charges, respectively.

#### 3.2.5. Drug Release Test

**Figure 7:**
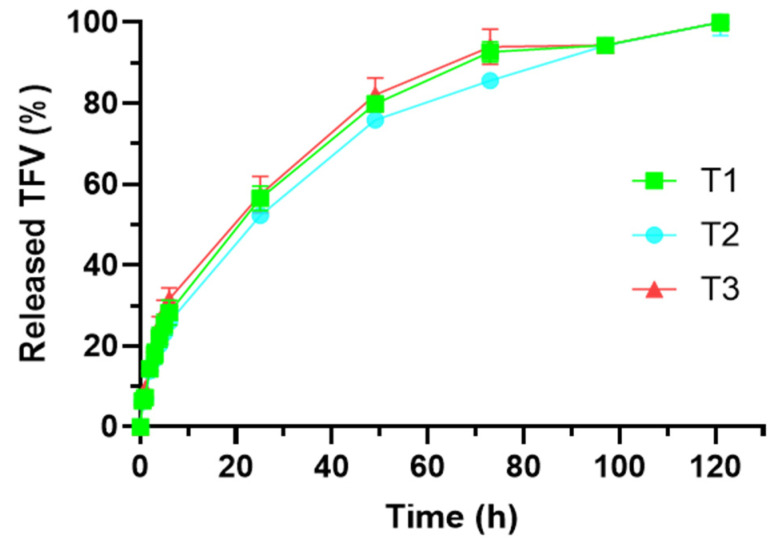
TFV release profiles in SVF of tablets manufactured including SiOC particles.

The tablets including the TFV-loaded particles were evaluated through drug release studies. Figure 7 includes the profiles obtained in this test. As it can be observed, all the tablets show similar drug release profiles, allowing a controlled release of TFV for 5 days.

There are no significant differences in TFV release according to the ratio of polymers included, since the f_2_ factor proved the similarity among them (Table 6). It can therefore be affirmed that the chitosan/HPMC ratio does not condition the drug release from the tablets containing the SiOC material. This finding was previously observed with TFV-loaded tablets based on the same polymers in the same proportions but including ACDP as the structuring agent instead of the inorganic particles. This is a positive finding, as it proves that both polymers maintain their individual ability to control the release of TFV, even with the incorporation of the SiOC particles [11]. Moreover, it reveals that the TFV loaded in the SiOC particles is able to be released to the environment in the presence of SVF, guaranteeing effective concentrations in the vagina.

The drug release profiles were fitted to three different mathematical models (Higuchi, Hopfenberg, and Korsmeyer–Peppas) to better understand the TFV release mechanism from the system (Table 7). As can be noted, all the tablets have a better fit to Higuchi kinetic, which means that diffusion is the mechanism that controls TFV release [39]. Theoretically, Higuchi kinetic does not apply to systems that undergo gelation [40]. However, there are references that confirm that the drug release in HPMC-based extended-release tablets that undergo gelation follows diffusional release. The prompt swelling of the tablet and the stability through several days of the gel formed—as observed in swelling studies—could explain that the release, once the tablet is swollen, is just conditioned by drug diffusion. As an alternative, we can determine the drug release mechanism based on the n value obtained when the drug release profiles are fitted to the kinetic of Korsmeyer–Peppas. A value under 0.45 would imply drug diffusion as the only mechanism controlling the release. This time, a value slightly over 0.45 is obtained for the three combinations, which implies that there is a combination of drug diffusion (the predominant process) and structural modification [41]. Thus, it is expected that, while the tablet swells, the modification of the system structure influences TFV release, but once it has captured a notable volume of water, the process that controls the release of the drug is diffusion. When comparing these tablets with the ones manufactured with ACDP instead of SiOC particles, it is observed that the value of *n* in Korsmeyer–Peppas model is lower when the particles are included [11]. This implies a main role of diffusion in drug release, which clearly can be associated to the lower swelling—and therefore, lower structural modification—observed for these tablets.

### 3.3. Characterization of Coated Tablets

The evaluation of the tablets including TFV-loaded SiOC particles, based on mixtures of HPMC and chitosan as matrix-forming polymers, allowed reaching several conclusions. At first, it has been proven that the SiOC particles comply with a double function. They serve as structural agent, giving as result a denser gel, with lower medium pore sizes that leads to less swollen tablets. This lower water capture implies that the system shows an improved mucoadhesion capability. In addition, the SiOC particles serve as a reservoir for TFV, since the drug loaded can be completely released to the medium.

However, another strategy that was studied to extend the release of the drug from the tablets is film coating with insoluble but permeable polymers. Eudragit^®^ RS, which is one of the most adhesive polymers in the Eudragit^®^ family [20], was used as coating polymer to ensure the adhesion of the system to the vaginal mucosa. Thus, the previously manufactured tablets were also studied—in terms of swelling and drug release—after their film coating.

#### 3.3.1. Swelling Test

**Figure 8:**
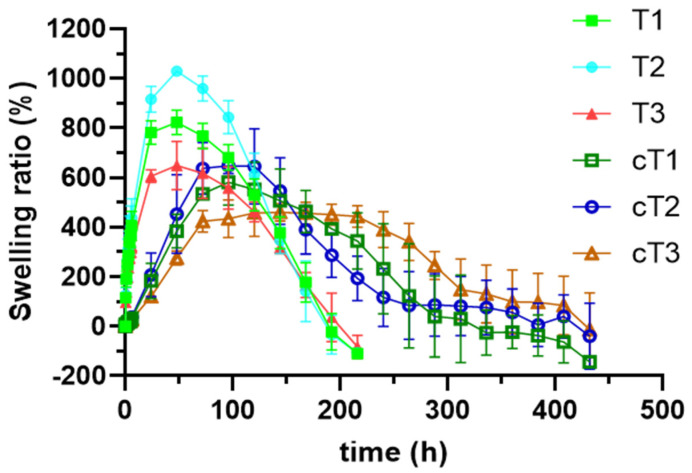
Swelling profiles resulting from the evaluation of the coated tablets in SVF, compared to the results obtained in uncoated tablets.

Swelling profiles obtained from the coated tablets are displayed in Figure 8. Although differences among them are not so evident as for uncoated tablets, cT2 reaches higher swelling values than cT1 and cT3, being the last one the batch with the lowest maximum swelling ratio. On that basis, it can be affirmed again that the higher the proportion of HPMC in the tablets, the higher their swelling. However, there are more notable differences when comparing coated and uncoated tablets. In coated tablets, the maximum swelling ratio is reduced to almost the half of the uncoated ones. Moreover, the time required to reach this maximum is also delayed—from 48 h for uncoated tablets to 96–120 h in the coated ones. This is related to the presence of the insoluble but permeable Eudragit^®^ RS film coating, that hinders the diffusion of fluid into the tablet—thus delaying the maximum—and holds the swellable polymers, which have a limited space to get swollen. This result was expectable, since Eudragit^®^ RS films have proven their inability to capture water when immersed in vaginal fluid [42]. The low amount of quaternary ammonium groups in this methacrylate derivative is responsible for its low permeability to aqueous fluids [43]. Undoubtedly, this modification in water capture behavior will have an influence on drug release from the coated tablets.

#### 3.3.2. Drug Release Test

**Figure 9:**
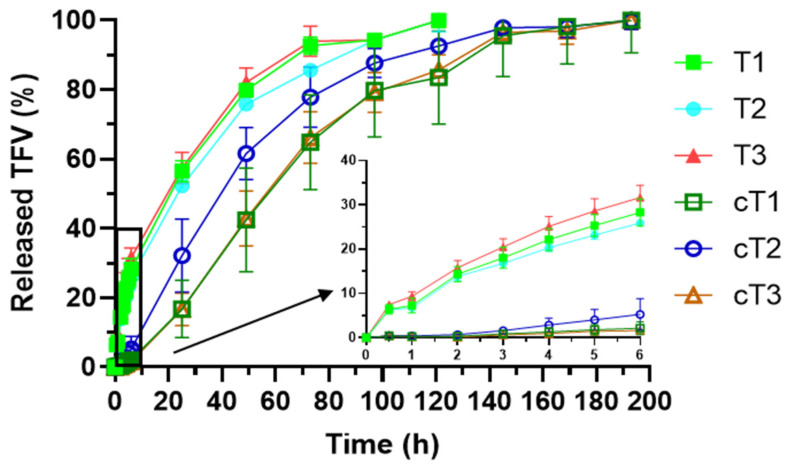
Drug release profiles from the formulated tablets.

Figure 9 includes the release profiles of all tablets developed (coated and uncoated). A controlled release of TFV for 8 days was achieved from the three batches of coated tablets. As it can be seen in the figure, cT1 and cT3 profiles are very similar, without significant differences between them at any point of the test. cT2 seems to release the drug faster than the previous batches but f_2_ pointed to similarity among the profiles of the three batches of coated tablets. Again, the chitosan/HPMC ratio does not influence the drug release from the formulations. Although the rate of TFV release is notably reduced by film coating (mainly in the firsts 6 h of test), the amounts of drug released after 30 min (approximately 0.25 mg) guarantee that the concentration of drug in the vagina would be significantly over the inhibitory concentration 50 (IC_50_) of TFV, that has been established between 1.08 and 1.22 µM [44].

Regarding the TFV release mechanism in these batches, when the drug release profiles were fitted to the previously mentioned kinetics, this time a better adjustment to the Hopfenberg model is obtained (Table 8). However, this model implies that drug release is controlled by heterogeneous erosion of the system, and diffusional resistance external to the matrix—in this case, the film coating—has no influence over the release of the drug. In the developed coated tablets, this assumption would imply that drug release depends on the erosion of the gel formed by HPMC and chitosan, being the Eudragit^®^ coating responsible for the delaying in the erosion of the gelled polymers, as observed in swelling studies. When analyzing the n values obtained in the adjustment to Korsmeyer–Peppas model, all values are over 0.89, which implies a “supercase II transport”—a mechanism that involves the structural modification of the system caused by the relaxation of the polymer swollen matrix [45]. Thus, the erosion of the HPMC/chitosan gel formed inside the Eudragit^®^ RS coating would be responsible for TFV release.

Finally, when comparing the coated tablets with the uncoated ones, a higher control over the release of the drug is clearly observed for coated batches regardless the chitosan/HPMC ratio, as confirmed by f_2_ (Table 9). This result was expectable, since there are references to the use of Eudragit^®^ RS for sustained release coating in pellets or tablets [46,47]. The low percentages of released TFV reached during the first hours of the test by the coated tablets should be highlighted. This behavior is responsible for the great differences in the release against the tablets without coating and allows to get a slightly delayed release, because there are no detectable drug levels in the medium until 2 h after the beginning of the test. It can be related to the coating-forming polymer and the drug release mechanism that has been described above. Prior to drug release, water diffusion through the Eudragit^®^ RS film coating and swelling of the HPMC/chitosan matrix are required; it is not until this occurs that TFV begins to be released. Subsequently, the drug release process in these systems is sustained for longer times than in uncoated tablets.

#### 3.3.3. Mucoadhesion Test

The ex vivo adhesion to vaginal mucosa of coated tablets was evaluated similarly to the uncoated ones. This time, all coated systems remained attached to vaginal mucosa for 72 h. It was expectable to have similar results between tablets with different composition, because the contact with the mucosa happens through the film coating—based on Eudragit^®^ RS and TEC—which is identical for all tablets. In this case, film coating is a disadvantage for the adhesion of the system, since the adhesion time is notably reduced compared to the uncoated ones. This is related to the excellent adhesive properties of the swellable polymers, HPMC and chitosan, and the fact that the adhesion provided by hydrogen bonds is more durable than the adhesion by electrostatic charges, which is responsible for the adhesion of Eudragit^®^ RS to the vaginal mucosa [21].

## 4. Conclusions

HPMC and chitosan-based vaginal tablets are able to form a gel in the presence of vaginal fluid, which allows the release of the antiretroviral drug TFV to be controlled. The drug can be loaded into APS-functionalized SiOC particles, which act as a reservoir of the active principle when included in the tablets. When both mechanisms for controlling drug release are combined in the same formulation, TFV release is sustained for 5 days. The inclusion of the SiOC particles in the tablets also modifies the microstructure obtained after the gelation of the system; it decreases the medium pore size and the total pore volume, and as a consequence the amount of vaginal fluid that is incorporated to the structure is notably reduced. The formation of this denser gel results in an improved mucoadhesion of the system to the vaginal mucosa, up to 6–7 days in ex vivo studies.

Film coating of the previously manufactured tablets with the methacrylic derivative Eudragit^®^ RS, plasticized with triethyl citrate, allows modifying the mechanism for TFV release. Thus, the coated tablets required water diffusion through the film coating to achieve HPMC and chitosan gelation, and the swelling of the coated system is lower and extended for longer times, which results in TFV controlled release for up to 7 days. This formulation could be an interesting strategy for the prevention of HIV-1 sexual transmission, since a single administration could provide effective drug levels in the vagina during a week. However, the film coating reduces the ex vivo mucoadhesion of the tablets, and this could hamper the retention of the system in the vagina. Therefore, further studies are required to evaluate the real in vivo retention time. The sustained drug release provided by film coating and the mucoadhesion obtained with the swellable polymers must be balanced to ensure the suitability of the developed system for this purpose. The administration of the developed vaginal tablets would be useful to prevent HIV infection although more assays should be carried out to confirm this point.

## Figures and Tables

**Table 1:** Composition of the tablets (mg/unit).

Tablet	Chitosan	HPMC	SiOC-TFV	PVP	MgSt
T1	145	145	75	27	3
T2	100	190	75	27	3
T3	190	100	75	27	3

**Table 2:** Composition of the coated tablets (mg/unit).

Coated Tablet	Chitosan	HPMC	SiOC-TFV	PVP	MgSt	Coat
cT1	145	145	75	27	3	39.5
cT2	100	190	75	27	3	39.5
cT3	190	100	75	27	3	39.5

**Table 3:** Properties of the tablets (mg/unit).

Tablet	Thickness (mm)	Diameter (mm)	Hardness (N)	Porosity (%)
T1	2.57 ± 0.02	13.04 ± 0.01	249.8 ± 9.1	2.02
T2	2.57 ± 0.03	13.02 ± 0.01	279.5 ± 16.0	3.46
T3	2.41 ± 0.01	12.98 ± 0.02	339.7 ± 5.9	1.01

**Table 4:** Pore volume (Vp), pore area (Sp), mean pore size (Dp), bulk and apparent densities (ρB, ρA), and porosity (P) of freeze-dried swollen tablets.

Tablet	Vp (cm^3^·g^−1^)	Sp (m^2^·g^−1^)	Dp (μm)	ρ_B_ (cm^3^·g^−1^)	ρ_A_ (cm^3^·g^−1^)	P (%)
T1	4.11	0.47	35.20	0.19	0.92	79
T2	5.20	0.70	29.95	0.16	0.98	84
T3	3.92	0.59	26.62	0.20	0.94	79

**Table 5:** Results from mucoadhesion test.

Tablet	Result
T1	Detachment at 144–168 h
T2	Detachment/erosion at 168 h
T3	Erosion at 144–168 h

**Table 6:** Similarity factor (f_2_) values for the release profiles obtained from reference and problem formulations.

Reference	Problem	f_2_
T1	T2	76.5
T1	T3	82.6
T2	T3	67.6

**Table 7:** Values obtained in the fit of TFV release profiles obtained from tablets to Higuchi, Hopfenberg and Korsmeyer–Peppas models. Values with the best fit (higher value of R^2^) are in bold.

Tablet	Higuchi		Hopfenberg		Korsmeyer–Peppas		
	K	R^2^	K	R^2^	K	n	R^2^
T1	0.113	**0.997**	0.010	0.980	0.093	0.57	0.984
T2	0.105	**0.996**	0.008	0.970	0.087	0.57	0.984
T3	0.118	**0.996**	0.011	0.961	0.110	0.54	0.987

**Table 8:** Values obtained in the fit of TFV release profiles obtained from coated tablets to Higuchi, Hopfenberg and Korsmeyer–Peppas models. Values with the best fit (higher value of R^2^) are in bold.

Tablet	Higuchi		Hopfenberg		Korsmeyer-Peppas		
	K	R^2^	K	R^2^	K	n	R^2^
cT1	0.085	0.955	0.005	**0.990**	0.003	1.17	0.933
cT2	0.100	0.969	0.007	**0.995**	0.005	1.20	0.961
cT3	0.086	0.954	0.006	**0.993**	0.003	1.23	0.892

**Table 9:** Similarity factor (f_2_) values for the release profiles obtained from reference and problem formulations. Comparisons with significant differences (f_2_ < 50) are in bold.

Reference	Problem	f_2_
T1	cT1	**32.5**
T1	cT1	**43.6**
T2	cT2	**31.1**

## Data Availability

Raw data are available upon request to the corresponding author.
